# Patient, study thyself

**DOI:** 10.1186/s12916-018-1216-2

**Published:** 2018-11-23

**Authors:** Paul Wicks

**Affiliations:** PatientsLikeMe, 160 2nd Street, Cambridge, MA 02142 USA

**Keywords:** Patient engagement, Medical informatics, Patient-reported outcomes, Smartphones, Machine learning

## Abstract

The past 15 years have seen the emergence of a new paradigm in medical research, namely of people living with medical conditions (whether patients, parents, or caregivers) using digital tools to conduct N-of-1 trials and scientifically grounded research on themselves, whilst using the Internet to form communities of like-minded individuals willing to self-experiment. Prominent examples can be found in amyotrophic lateral sclerosis/motor neurone disease (the ‘lithium study’ on PatientsLikeMe), Parkinson’s disease (‘digital patient’ Sara Riggare), and diabetes (the ‘open artificial pancreas’ of the #WeAreNotWaiting movement). Through transparency, data sharing, open source code, and publication in the peer-reviewed scientific literature, such activities conform to expected scientific conventions. However, other conventions, such as ethical oversight, regulation, professionalization, and the ability to translate this new form of relatively biased data into generalizable decisions, remain challenged. While critics worry such participant-led research merely muddies the waters of high-quality medical research and exposes patients to new harms, the potential is there to enroll millions of active minds in unravelling the wicked problems of complex medical disorders that degrade the human health span.

## Background

At the time of *BMC Medicine*’s launch 15 years ago, I was conducting what I thought was fairly ‘high-tech’ neuropsychology research for my PhD. Each week, I would drive out to the homes of people living with amyotrophic lateral sclerosis (ALS) to administer computerized psychological tests and invite them to our neuroimaging unit to have MRI and PET brain scans [1]. Since then, the most visible changes in medical research have been technological; for example, the heavy 486 laptops and the stack of inch-thick paper test batteries that I wheeled around in a 22 kg suitcase would now work more reliably as software apps on a 0.5 kg iPad [2]. However, I do not believe that the consumer electronics revolution of smartphones, tablets, and social media is the most profound change to have taken place during that time.

## Patients are doing it for themselves

Instead, I think the largest shift in the past 15 years has been social, wherein patients, caregivers, parents, and family members have realized that ‘research’ is not just something that professionals do to them when they venture forth from their ivory towers, but that science and research are a set of tools and thinking methods that can be applied by anyone. My own wakeup call arrived in 2002, when I volunteered to take over as webmaster for an online community of people with ALS in the UK, called BUILD-UK [3]. As I was studying the supposedly rare issue of dementia in ALS, I was relentlessly grilled by patients rightfully asking why I was studying a potentially rare consequence of the disease that would take years to reap insights when patients were dying right now. Could the money not be better deployed towards clinical trials? I was surprised and impressed at the way they dissected clinical trial protocols, posed many of the same research questions I wondered about at conferences, and actually generated solutions that could immediately help other patients like them, rather than waiting for a peer-reviewed article to appear in print several years later. Most research professionals I spoke to seemed to merely tolerate ‘lay people’ having these discussions. However, not until one patient requested knowledge of their trial group allocation and actually had their study medication sent to a lab for testing, were they taken seriously [4].

## Participant-led clinical trials and trial unblinding in ALS

In early 2008, another group of exasperated ALS patients initiated their own participant-led trial of the drug lithium carbonate, which had been touted by some over-enthusiastic professional researchers as a near-cure for this dreadful disease [5]. Within 6 months, a patient in Brazil and a caregiver in the United States had rallied over 160 patients (10 times the original paper’s sample size) to begin taking the drug off-label whilst systematically reporting their dosage, patient reported outcomes, side effects, and weight as well as running their own statistical analyses publicly on a weekly basis. All the data was reported online so that participants, and other members of the community, could see the data coming through in real-time. Some patients even recorded pre-/post-exposure videos or tried to conduct objective tests of muscle strength. Building on internal analyses by the patients, the research team at PatientsLikeMe shared our own analysis at the International ALS/Motor Neurone Disease Symposium just 9 months later, wherein we found that lithium did not work [6]; we later shared our data for others to check [7]. At least five subsequent randomized control trials by academic researchers confirmed these findings [8]. However, critics of the participant-led study pointed to the lack of a traditional protocol, lack of blinding, and questionable ethics [9]. When a later tranche of the ALS community used the same approach to begin attempting to systematically unblind themselves at scale in blinded randomized control trials, alarm bells really started ringing [10]. Regardless of what researchers thought, patients were making their feelings clear – they did not have the time to wait for answers. I knew that to be true since, tragically, all participants in my PhD study had died by the time the results were formally published and it is only today that the implications are making their way into clinical practice [11].

## Who can grant you permission to experiment on yourself?

When Swedish engineer Sara Riggare was diagnosed with Parkinson’s disease aged 32, she did not just accept her doctor’s advice – after all, she only saw him for approximately 1 hour annually and was responsible for her own self-care for the remaining 8765 hours. She enrolled herself for a PhD in Parkinson’s disease at the Karolinska Institute and put her training to use. Sara devised objective and quantitative measures of how her dopaminergic system was functioning and interacting with her medications using smartphone technology to measure her finger tapping speed [12]. She has published peer-reviewed studies, including a survey of Swedish Parkinson’s patients on how they self-manage [13] and an N-of-1 study using nicotine through an electronic cigarette to modulate symptoms and side effects [14]. Sara’s pioneering work is interesting enough that she has even been a featured ‘exhibit’ at the British Science Museum and a celebrated inspiration to patients and researchers alike; yet, 6 years after starting her doctoral program, she hit an unexpected roadblock. Because she had neither sought nor been granted ethical approval to experiment on herself, the doctoral dissertation committee declined her application to defend her PhD thesis (http://www.riggare.se/2018/09/16/ethics-and-phd/#more-1979). In practice, her studies had merely involved tapping her smartphone over a few days and smoking an e-cigarette, both of her own volition. Whereas ethical standards were constructed on a national level and centered around medical and educational institutions, the self-studying patient violated many of these assumptions. In one study, Sara tested her tapping speed while on an airplane crossing international waters – could Swedish law still apply? Such ‘edge cases’ are essential in adapting our existing frameworks while being cautious not to erode key protections for potentially vulnerable people [15].

## Hacking diabetes

However, participant-led research does not just end with a published peer-reviewed journal article. In 2014, a ground-breaking initiative was led by people living with diabetes in conjunction with parents of children living with the condition. They united under the hashtag #WeAreNotWaiting and used their technical abilities to construct new ‘open loop’ systems – out of jury-rigged configurations – in their continuous glucose monitors, s-martphones, wearable devices, and older insulin pumps hacked to receive instructions from crowd-sourced software code [16]. While early results have been positive, including a high degree of safety and an impressive effect on HbA1c levels among this self-selecting group, the regulatory issues have been understandably complex [17]. The US Food and Drug Administration has engaged with the Nightscout group and so long as patients are building their own systems, rather than distributing them for commercial use, they appear exempt from regulation [17]. While it might be safer for an experienced builder to construct a closed loop system for another patient, this would bring with it unbearable liability and risk [18]. Thus, worldwide, small teams of people meet for ‘DIY build parties’ to discuss checklists and construct their own open loop systems. Once online, people share their experiences, data, and code to facilitate others to participate (https://www.diabettech.com/).

## Conclusions

The history of medicine encompasses much praise for doctors and scientists who experimented on themselves – Dr. Werner Forssmann won the Nobel prize after performing the first heart catheterization procedure on himself, and Dr. Barry Marshall won the prize for giving himself a stomach ulcer by swallowing a petri dish full of bacteria (https://scienceofparkinsons.com/2017/10/30/one/#more-47943). Perhaps it is not so farfetched to think that in the next 15 years we might see a patient with no formal scientific or medical training win a Nobel prize for insights that arose from studying themselves and their peers. Perhaps one day the mantra will be “Patient, cure thyself”.
